# Deep versus shallow sources of CO_2_ and Rn from a multi-parametric approach: the case of the Nisyros caldera (Aegean Arc, Greece)

**DOI:** 10.1038/s41598-020-70114-x

**Published:** 2020-08-13

**Authors:** Giulio Bini, Giovanni Chiodini, Carlo Lucchetti, Piergiorgio Moschini, Stefano Caliro, Silvio Mollo, Jacopo Selva, Paola Tuccimei, Gianfranco Galli, Olivier Bachmann

**Affiliations:** 1 Department of Earth Sciences, Institute of Geochemistry and Petrology, ETH Zurich, 8092 Zurich, Switzerland; 2 Istituto Nazionale di Geofisica e Vulcanologia, Sezione di Bologna, 40128 Bologna, Italy; 3 Dipartimento di Scienze della Terra, Sapienza-Università di Roma, 00185 Rome, Italy; 4 Istituto Nazionale di Geofisica e Vulcanologia, Osservatorio Vesuviano, Sezione di Napoli, 80124 Napoli, Italy; 5 Istituto Nazionale di Geofisica e Vulcanologia, Sezione di Roma 1, 00143 Rome, Italy; 6 Dipartimento di Scienze, Università Roma Tre, 00146 Rome, Italy

**Keywords:** Geochemistry, Volcanology

## Abstract

Estimating the quantity of CO_2_ diffusively emitted from the Earth’s surface has important implications for volcanic surveillance and global atmospheric CO_2_ budgets. However, the identification and quantification of non-hydrothermal contributions to CO_2_ release can be ambiguous. Here, we describe a multi-parametric approach employed at the Nisyros caldera, Aegean Arc, Greece, to assess the relative influence of deep and shallow gases released from the soil. In April 2019, we measured diffuse soil surface CO_2_ fluxes, together with their carbon isotope compositions, and at a depth of 80 cm, the CO_2_ concentration, soil temperature, and the activities of radon and thoron. The contributions of deep CO_2_ and biogenic CO_2_ fluxes were distinguished on the basis of their carbon isotope compositions. A Principal Component Analysis (PCA), performed on the measured parameters, effectively discriminates between a deep- and a shallow degassing component. The total CO_2_ output estimated from a relatively small testing area was two times higher with respect to that observed in a previous survey (October 2018). The difference is ascribed to variation in the soil biogenic CO_2_ production, that was high in April 2019 (a wet period) and low or absent in October 2018 (a dry period). Accounting for seasonal biogenic activity is therefore critical in monitoring and quantifying CO_2_ emissions in volcanic areas, because they can partially- or completely overwhelm the volcanic-hydrothermal signal.

## Introduction

The emission of volcanic-hydrothermal fluids from fumaroles and soil diffuse degassing structures (DDS) are prevalent forms of thermal energy release in quiescent volcanoes^1,2^, and their monitoring is of primary importance in understanding volcanic activity^3-6^. The amount of CO_2_ emitted by volcanic DDS is thought to be, globally, a relevant contributor (likely the most important) to the CO_2_ budget from volcanic activity to the atmosphere^7,8^. However, the uncertainties in determining the amount of the volcanic diffuse CO_2_ emission are significant, as biological activity can also produce abundant CO_2_. In fact, over the last 20 years, the definition and characterization of the diffuse degassing processes has been based, with a few exceptions, only on CO_2_ flux measurements without differentiating between their possibly disparate deep- (i.e., volcanic-hydrothermal) or shallow (i.e., biogenic) sources. Coupling CO_2_ flux measurements with other parameters collected from the surface or within the soil in volcanic areas (e.g. ^2,9,10^), can be crucial to better decipher the actual fraction of gas emitted from magmatic-hydrothermal systems. This approach is of fundamental importance in cases where the statistical partitioning^11^ and subsequent removal from the total CO_2_ output of non-hydrothermal CO_2_ flux (of biogenic origin) is not easily applicable or ambiguous. Ultimately, a multi-parametric strategy circumvents over-interpretation in the extent and the amount of deep degassing estimated for active volcanoes worldwide.

In addition to monitoring of CO_2_ in volcanic areas, the determination of radon (^222^Rn) and thoron (^220^Rn)—two radiogenic nuclides produced within the crust by the radioactive decay of radium progenitors in the U-Th decay series (ref.^12^ and refs therein)—in the soil gas phase may help differentiating shallow and deep sources of gas^9,13^. Owing to the very different half-lives of ^222^Rn (3.82 d) and ^220^Rn (55.6 s), their ratio can be a proxy for the depth at which they are released. In absence of any preferential flow pathways, such as fractures and faults, radon gas diffuses and disperses slowly through the rock-soil pores, covering only very short distances from its source^14^. Consequently, ^222^Rn-^220^Rn activities detected in the subsurface are mostly related to the contribution of shallow gas production by U-Th-bearing minerals in the rock-soil matrix. However, in active volcanic areas and fault zones, the relatively fast advective transport of fluids along permeable structures may mobilize the radon gas produced in the crust for longer distances^15,16^. According to this scenario, the gas measured from volcanic soil may be interpreted as a mixture between two different contributions^9^: (1) shallow radon gas production (i.e., low ^222^Rn/^220^Rn ratio) within an undisturbed soil and (2) deep radon gas production (i.e., high ^222^Rn/^220^Rn ratio) with transport towards the surface in the presence of carrier gases (e.g., CO_2_).

**Figure 1:**
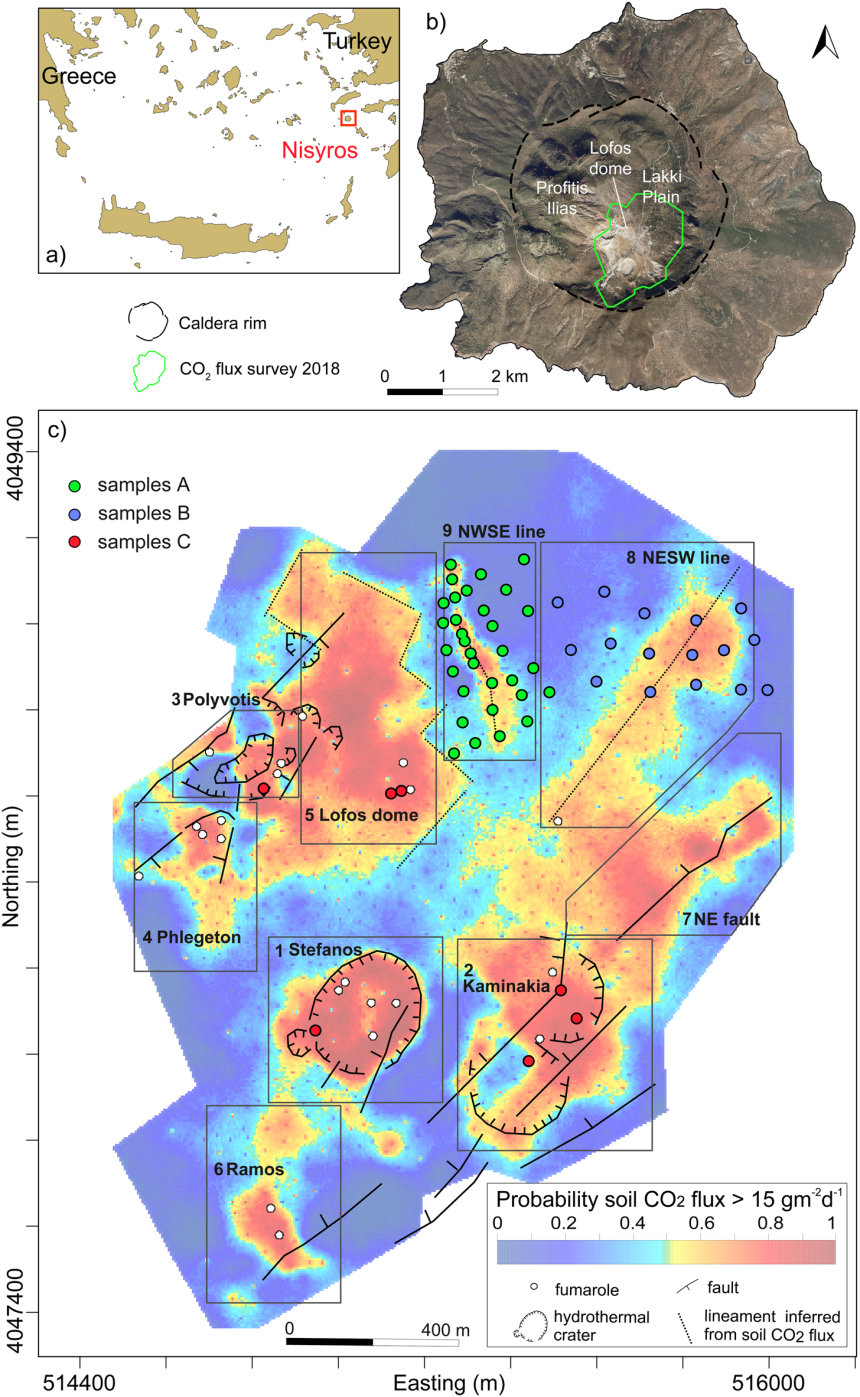
(**a**) Location of Nisyros and (**b**) extent of the caldera and of the CO_2_ flux survey of October 2018^19^. The map was generated using the open source QGIS software (available at https://www.qgis.org/it/site/) using the Bing Aerial base map. (c) Probability map of the CO_2_ flux measured during October 2018 (redrawn after ref.^19^). The 9 diffuse degassing structures (DDS) are defined by solid line perimeters. DDS 7, 8, and 9 cover the southern part of the Lakki plain, while the DDS 1–6 extend over the hydrothermal area of the caldera (white circles indicate the fumarolic vents). Samples A, B, and C are reported as green, blue and red circles, respectively. Easting and northing coordinates refer to the WGS 84/UTM zone 35 S.

Here, we report and discuss the results obtained by a multi-parametric survey, with the aim of unequivocally determining the sources of the gases measured from the soil of the Nisyros caldera (Aegean Arc, Greece; Fig. 1a, b), selected as a test site. The Nisyros caldera is one of the earliest volcanoes at which soil CO_2_ fluxes were measured^17^ and where detailed maps of the degassing structures exist^18,19^. A recent CO_2_ flux survey has documented that the soil CO_2_ emission is controlled by nine different DDS (Fig. 1c; ref.^19^), which correspond to the fracture network of the caldera and to the hydrothermal craters. From the 1st to the 9th of April 2019, we monitored emissions at 55 locations (Fig. 1c): (1) 32 sampling points located at the DDS 9 (samples A); (2) 16 sampling points located at the east of DDS 9 (samples B); (3) 7 sampling points randomly located at the hydrothermal craters (samples C).

Among these sites, we focus more specifically on samples A, which extend over a DDS located at the southwestern edge of the Lakki plain and adjacent to the Lofos dome (DDS 9 in Fig. 1c). In the samples A area, anomalous soil CO_2_ emission is related to a NW–SE lineament, which probably corresponds to the buried tip of a 2-km-long NW–SE fault cutting the Profitis Ilias dome^18^. This fault was active during the last seismic swarm recorded at Nisyros in 1996–1997^20^. Due to sedimentary cover and the superposition of vegetated soils, there is no visible surface evidence of hydrothermal outgassing (e.g., argillic alteration, sulfur deposition or fumarolic vents).

Measurements of different parameters were performed during a wet season. We selected H_2_S-free sites to avoid the prolonged exposure of the RAD7 radon detector to sulfur-bearing gas species. The sampling strategy adopted for each site consisted of measurements of the soil CO_2_ flux (in g m^−2^ d^−1^), determination of the carbon isotopic composition in the CO_2_ efflux from the ground (*δ*^13^C_CO2_ in ‰ vs. V-PDB), as well as measurements of soil temperature (*T* in °C), CO_2_ concentration (C_CO2_ in vol%) and ^222^Rn (radon) and ^220^Rn (thoron) activities (in kBq m^−3^) at 80 cm of soil depth (Table 1). In addition, we measured the soil permeability (*k* in m^2^; see “Methods”), which displayed medium-to-high values. Finally, we sampled and analyzed the *δ*^13^C_CO2_ of the main fumaroles, and specific laboratory experiments were designed to characterize Rn exhalation from soils and rocks in the area of interest (see “Methods”).

**Table 1:** Gas measurements at Nisyros caldera at the surface or at 80 cm of soil depth.

n	Date	CO_2_ flux	*deep*CO_2_ flux	*bio*CO_2_ flux	*δ*^13^C_CO2,e_	C_CO2_	*T*	*k*	^222^Rn	^220^Rn	^222^Rn/^220^Rn	*x*	*y*
		g m^−2^ d^−1^	g m^−2^ d^−1^	g m^−2^ d^−1^	‰ vs. PDB	%	°C	m^2^	kBq m^−3^	kBq m^−3^	–	m	m
A1	01/04/19	26.7	0.0	26.7	− 29.7	3.8	22.3	1.61E−11	6.0	8.9	0.7	515,286	4,048,773
A2	01/04/19	46.3	42.0	4.4	− 3.5	9.8	28.1	2.01E−11	9.4	5.9	1.6	515,356	4,048,802
A3	01/04/19	31.8	3.1	28.7	− 24.6	9.2	19.4	2.67E−11	9.1	7.3	1.2	515,424	4,048,836
A4	01/04/19	8.4	2.9	5.5	− 18.0	4.0	15.6	3.99E−11	22.2	12.1	1.8	515,451	4,048,898
A5	01/04/19	26.0	0.0	26.0	− 26.5	9.0	20.6	2.01E−11	20.7	12.2	1.7	515,402	4,048,870
A6	01/04/19	51.3	29.8	21.5	− 12.0	11.6	23.6	2.67E−11	14.2	10.4	1.4	515,355	4,048,864
A7	01/04/19	13.5	0.0	13.5	− 30.7	1.6	16.5	3.99E−11	5.6	10.6	0.5	515,288	4,048,845
A8	01/04/19	13.0	2.5	10.6	− 22.3	1.6	16.5	2.67E−11	6.1	4.4	1.4	515,263	4,048,891
A9	02/04/19	12.9	0.0	12.9	− 26.2	2.8	17.3	2.22E−12	9.8	5.2	1.9	515,489	4,048,842
A10	03/04/19	73.6	28.7	44.8	− 17.0	31.0	30.6	3.99E−11	58.2	11.6	5.0	515,311	4,048,909
A11	03/04/19	26.2	0.0	26.2	− 26.3	6.8	20.0	3.99E−11	20.0	9.4	2.1	515,379	4,048,938
A12	03/04/19	13.4	0.0	13.4	− 27.9	2.0	17.4	2.67E−11	11.9	14.9	0.8	515,356	4,048,996
A13	03/04/19	185.3	176.7	8.6	− 2.2	42.0	49.4	2.67E−11	97.1	12.8	7.6	515,291	4,048,961
A14	04/04/19	10.3	0.0	10.3	− 27.3	2.8	19.0	3.99E−11	6.7	6.1	1.1	515,248	4,048,940
A15	04/04/19	1.8	0.0	1.8	− 33.1	0.4	17.8	1.16E−11	23.9	19.6	1.2	515,335	4,049,032
A16	04/04/19	98.4	81.6	16.8	− 5.5	26.0	29.6	3.99E−11	40.6	14.1	2.9	515,270	4,049,010
A17	04/04/19	23.8	1.3	22.5	− 25.7	5.0	19.3	3.99E−11	16.6	15.1	1.1	515,240	4,049,003
A18	04/04/19	8.8	1.3	7.5	− 23.3	4.0	18.8	7.95E−11	12.4	15.6	0.8	515,241	4,049,049
A19	04/04/19	34.2	13.4	20.8	− 16.9	8.8	21.1	2.67E−11	13.8	17.4	0.8	515,268	4,049,062
A20	04/04/19	89.3	60.6	28.7	− 9.4	20.5	34.0	2.67E−11	22.2	14.0	1.6	515,261	4,049,104
A21	04/04/19	16.9	0.0	16.9	− 27.2	1.8	19.7	2.67E−11	10.6	17.9	0.6	515,295	4,049,078
A22	05/04/19	85.6	55.3	30.3	− 10.3	19.0	22.3	2.01E−11	27.9	17.1	1.6	515,258	4,049,138
A23	05/04/19	8.2	0.0	8.2	− 25.9	1.2	18.5	3.99E−11	9.4	17.1	0.6	515,328	4,049,116
A24	05/04/19	159.1	141.2	17.9	− 3.9	49.0	47.9	7.95E−11	81.7	14.9	5.5	515,284	4,048,978
A25	05/04/19	110.3	107.0	3.3	− 1.8	43.0	47.2	7.95E−11	126.5	14.0	9.0	515,304	4,048,933
A26	08/04/19	89.8	33.2	56.6	− 17.5	4.8	21.0	2.67E−11	8.3	5.3	1.6	515,266	4,048,701
A27	08/04/19	6.4	0.0	6.4	− 29.3	1.6	17.0	2.67E−11	17.6	7.1	2.5	515,315	4,048,725
A28	08/04/19	132.3	112.3	19.9	− 4.9	19.5	29.1	1.61E−11	19.8	5.9	3.3	515,372	4,048,740
A29	08/04/19	15.4	6.1	9.2	− 16.7	6.2	20.8	7.95E−11	19.8	6.8	2.9	515,439	4,049,031
A30	08/04/19	7.2	0.0	7.2	− 27.3	4.2	16.3	1.12E−12	24.5	16.2	1.5	515,422	4,049,041
A31	08/04/19	18.6	0.0	18.6	− 27.9	2.0	14.9	2.67E−11	25.5	10.8	2.4	515,387	4,049,080
A32	08/04/19	17.7	0.0	17.7	− 28.8	2.6	15.8	1.61E−11	25.7	12.2	2.1	515,429	4,049,151
B1	02/04/19	2.0	0.0	2.0	− 26.3	6.2	15.3	2.01E−11	17.9	3.0	5.9	515,599	4,048,868
B2	02/04/19	77.8	19.7	58.1	− 20.6	10.2	16.9	3.99E−11	22.2	7.0	3.1	515,725	4,048,844
B3	02/04/19	20.5	6.6	14.0	− 18.8	8.2	16.5	6.84E−12	34.3	10.7	3.2	515,832	4,048,860
B4	02/04/19	26.5	0.0	26.5	− 28.4	3.0	15.2	1.16E−11	18.0	29.7	0.6	515,936	4,048,849
B5	02/04/19	8.1	0.0	8.1	− 27.9	10.4	15.8	2.01E−11	19.1	9.7	2.0	515,998	4,048,848
B6	02/04/19	27.6	4.7	22.9	− 22.8	3.8	16.2	3.99E−11	10.2	9.9	1.0	515,967	4,048,964
B7	02/04/19	42.7	18.3	24.4	− 16.0	15.0	15.9	3.99E−11	21.8	7.0	3.1	515,897	4,048,940
B8	02/04/19	43.9	24.9	19.0	− 12.3	11.2	14.8	3.99E−11	17.2	6.6	2.6	515,822	4,048,930
B9	02/04/19	60.8	11.0	49.8	− 22.5	9.0	15.6	2.01E−11	17.0	7.0	2.4	515,721	4,048,932
B10	02/04/19	21.5	0.0	21.5	− 27.8	10.2	12.9	4.64E−12	37.8	7.9	4.8	515,632	4,048,956
B11	03/04/19	11.7	0.0	11.7	− 27.2	2.2	15.8	1.01E−11	10.7	5.3	2.0	515,539	4,048,941
B12	03/04/19	20.8	0.0	20.8	− 28.1	2.4	17.0	1.49E−12	7.3	12.1	0.6	515,508	4,049,051
B13	03/04/19	9.2	2.1	7.1	− 21.3	4.2	19.5	8.16E−12	25.4	10.4	2.4	515,618	4,049,076
B14	03/04/19	12.0	6.0	6.0	− 14.1	4.6	18.2	1.61E−11	21.0	6.3	3.3	515,711	4,049,026
B15	03/04/19	91.2	11.1	80.1	− 24.0	7.6	15.9	1.61E−11	18.4	10.0	1.8	515,832	4,049,009
B16	03/04/19	20.5	0.0	20.5	− 27.8	1.0	17.6	7.95E−11	4.3	15.1	0.3	515,937	4,049,038
C1	04/04/19	142.6	142.6	0.0	− 1.1	43.0	81.3	7.95E−11	9.8	0.3	29.6	515,142	4,048,613
C2	04/04/19	79.6	79.6	0.0	− 0.1	27.0	72.1	1.16E−11	36.4	2.8	13.2	515,118	4,048,607
C3	05/04/19	122.4	113.9	8.4	− 2.8	52.0	63.9	4.20E−12	12.4	0.5	27.3	515,516	4,048,152
C4	05/04/19	187.1	187.1	0.0	− 0.9	30.0	38.6	3.99E−11	10.1	0.3	30.0	515,553	4,048,087
C5	05/04/19	208.6	208.6	0.0	− 0.7	42.0	55.3	2.67E−11	40.9	0.9	43.9	515,440	4,047,988
C6	05/04/19	75.7	75.7	0.0	− 1.5	30.0	36.4	5.52E−12	11.1	0.2	48.3	514,943	4,048,059
C7	09/04/19	188.8	180.7	8.2	− 2.1	29.0	37.1	3.99E−11	59.1	1.9	30.5	514,820	4,048,618

Samples A, B, and C refer to DDS 9, the eastward area of DDS 9 and the hydrothermal craters area, respectively. Easting and northing coordinates (*x* and *y*, respectively) refer to the WGS 84/UTM zone 35 S. See the text for the meaning of the different variables.

The aim of the study is to clearly characterize the deep degassing signal coming from the magmatic-hydrothermal system as recorded by gases present in and emanating from the soil. In order to achieve this objective, we employ an approach based on the carbon isotopic signature in the CO_2_ efflux to quantitatively subdivide the measured CO_2_ fluxes (*meas*CO_2_ flux) into a fraction of magmatic-hydrothermal origin (*deep*CO_2_ flux) and a fraction derived from shallow biogenic production (*bio*CO_2_ flux) active in the soil. To simplify data interpretation, we apply Principal Component Analysis (PCA) on the entire multi-parametric data set.

## Results and discussion

**Figure 2:**
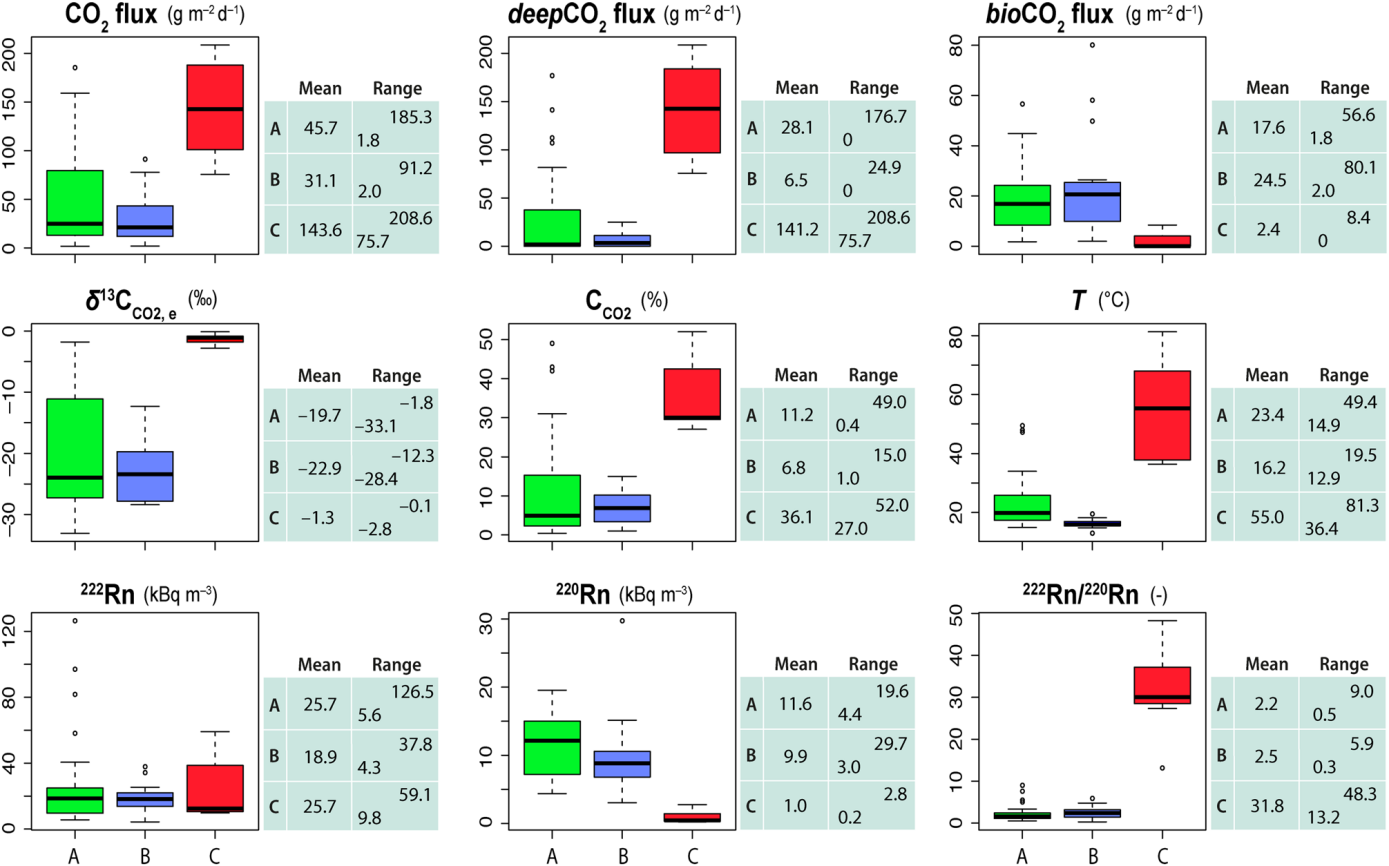
Boxplots and tables of the geochemical parameters (CO_2_ flux, *δ*^13^C_CO2,e_, C_CO2_, *T*, ^222^Rn, ^220^Rn, and ^222^Rn/^220^Rn ratio) measured at Nisyros caldera, summarizing the descriptive statistic of the data. Boxplots of the computed variables *deep*CO_2_ flux and *bio*CO_2_ flux are also shown. Samples A, B and C are marked with green, blue and red colors, respectively.

Results from our multi-parametric survey are listed in Table 1 and are summarized in the boxplots of Fig. 2, which describe the statistics of the data. In the following subsections, we briefly discuss the significance of each measured parameter in terms of its shallow versus deep origin, and we perform a multivariate analysis.

### Measured parameters

#### Soil temperature and CO_2_ concentration

**Figure 3:**
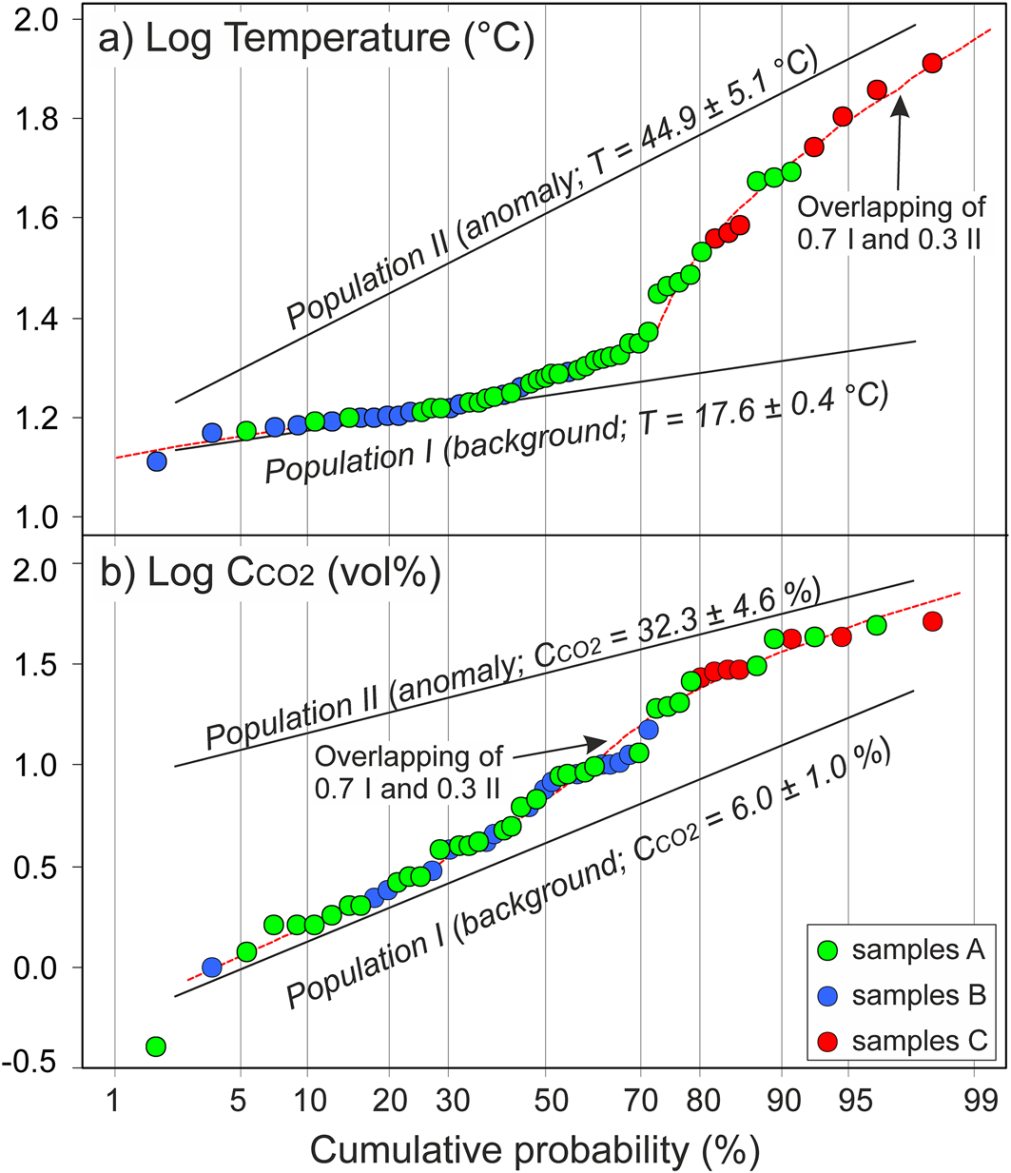
Probability plots of (**a**) soil temperatures and (**b**) soil CO_2_ concentrations at 80 cm depth. Both the plots define the overlapping of 0.7 population I (background) and 0.3 population II (anomaly). The anomalies refer to all the samples C (hydrothermal craters) and to some of the samples A (DDS 9).

Values of soil *T* and C_CO2_ range over large intervals (from 12.9 to 81.3 °C and from 0.4 to 52%, respectively; Table 1; Fig. 2), noting the different processes controlling each of these variables. Such processes were investigated applying the graphical statistical approach (GSA^11^, see “Methods”), that entails analyzing the distribution of the data in log-probability plots, where a normal population delineates a straight line, while *n* normal overlapping populations define curves characterized by *n*−1 inflection points. Both *T* and C_CO2_ data define curves characterized by one inflection point (Fig. 3), indicating the overlapping of background (population I in Fig. 3) and anomalous values (population II in Fig. 3).

The means of the background *T* and C_CO2_ populations (population I) were estimated at 17.6 °C ± 0.4 °C and 6.0% ± 1%, respectively, while the means of the anomalous *T* and C_CO2_ populations (population II) at 44.9 °C ± 5.1 °C and 32.3% ± 4.6%, respectively (for details on the calculations see “Methods”). It is worth noting that in both cases (*T* and C_CO2_) the anomalous values represent 30% of the measurements. This coincidence is not surprising, because both the anomalies are generated by the subsurface condensation of vapors rising from the hydrothermal system located at depth in the Lakki plain (ref. ^19^ and refs therein). In detail, the anomalously high temperatures reflect the latent heat of condensation, while the high C_CO2_ are caused by the CO_2_ contents of the original hydrothermal vapors.

#### Diffuse CO_2_ fluxes from biogenic and deep carbon sources

**Figure 4:**
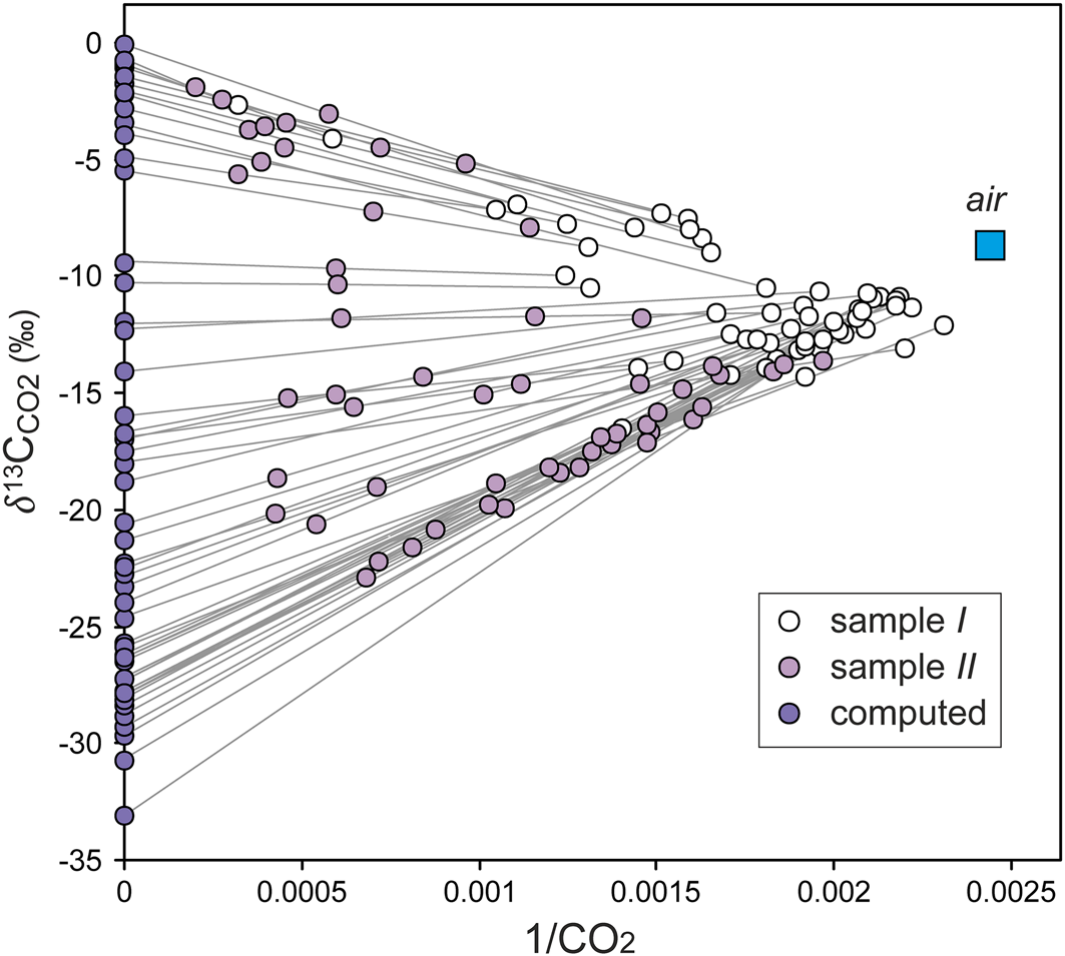
*δ*^13^C_CO2_ versus 1/CO_2_ plot. The concentration of CO_2_ is expressed as ppm by volume (i.e., pure CO_2_ corresponds to 1/CO_2_ = 0). The isotopic composition of the CO_2_ efflux (*δ*^13^C_CO2__,__e_; see the violet circles) was computed as the y intercept at x = 0 of the straight lines connecting the first (*δ*^13^C_CO2,I_ − C_CO2,I_) and second (*δ*^13^C_CO2__,__II_ − C_CO2,II_) samples. See the text for further explanation.

During each CO_2_ flux measurement with the accumulation chamber (AC; see “Methods”), we collected two samples of gas at different CO_2_ concentration to analyze both *δ*^13^C_CO2_ and C_CO2_ in laboratory (see “Methods”). Each couple of *δ*^13^C_CO2_ − C_CO2_ (*δ*^13^C_CO2,I_ − C_CO2,I_, *δ*^13^C_CO2,II_ − C_CO2,II_; Table S1) plotted in the *δ*^13^C_CO2_ versus 1/C_CO2_ diagram (Fig. 4), defines a mixing line between the CO_2_ present in the chamber at the time of the first measurement, and the CO_2_ entering the chamber during the interval of time between the two measurements (i.e., the soil CO_2_ efflux). The isotopic composition of each CO_2_ efflux (*δ*^13^C_CO2,e_, the gas entering the AC) has been computed as the intercept at 1/CO_2_ = 0 (i.e., pure CO_2_) of the straight line determined by the corresponding couple *δ*^13^C_CO2,I_ − C_CO2,I_, *δ*^13^C_CO2,II_ − C_CO2,II_.

**Figure 5:**
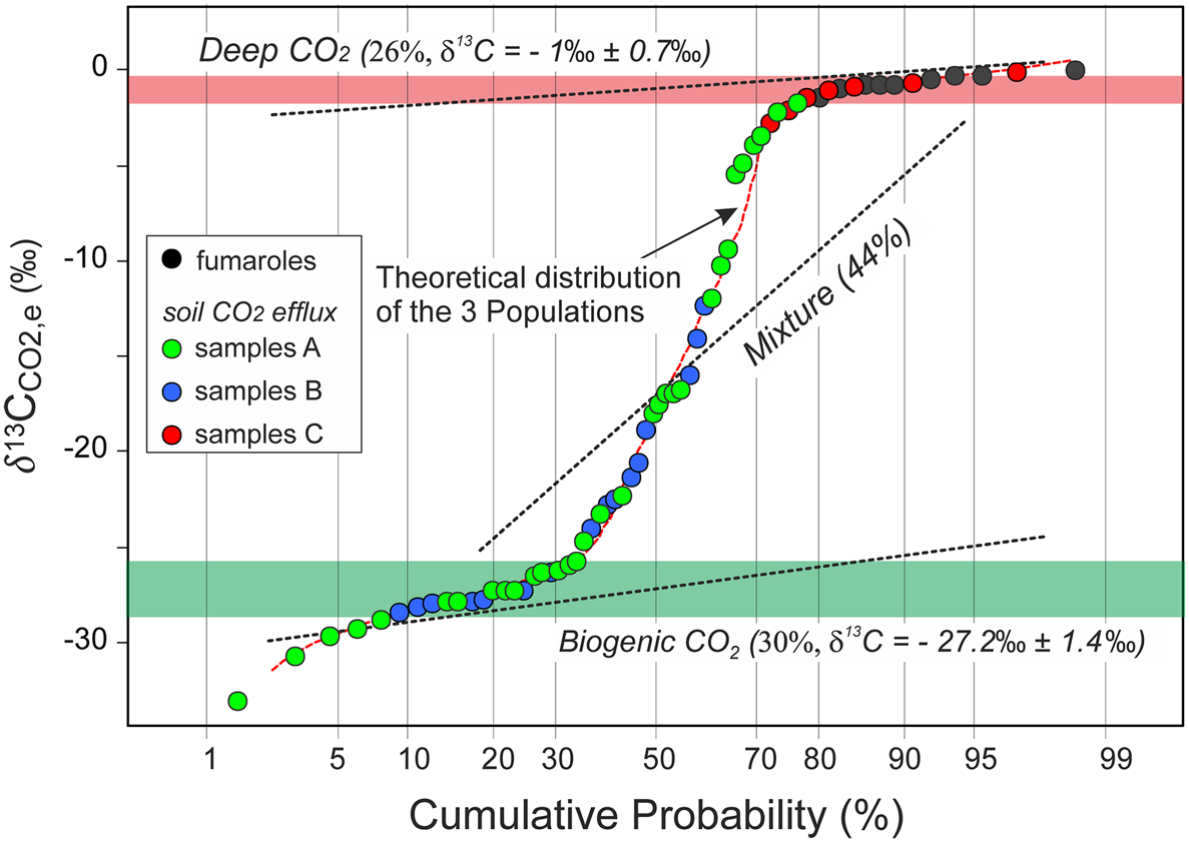
Probability plot of the carbon isotopic composition of the soil CO_2_ efflux and fumarolic CO_2_. The green and the red fields indicate the mean carbon isotopic composition ± 1σ of both biogenic and deep CO_2_ populations.

The *δ*^13^C_CO2,e_ of the measured gas was used to assess the relative contributions of the *bio*CO_2_ flux active in the soil and the *deep*CO_2_ flux coming from the hydrothermal system to the *meas*CO_2_ flux. The multi-step computation begins by partitioning the statistical distribution of the *δ*^13^C_CO2,e_ values, applying the GSA approach^11,21^ (see “Methods”). Values of *δ*^13^C_CO2,e_ plotted in the probability diagram (Fig. 5), define three distinct populations. About 30% of the samples belong to the biogenic CO_2_ population with the lighter values of *δ*^13^C_CO2,e_ (*δ*^13^C_CO2, *bio*_ = − 27.2 ± 1.4‰), typical of the biogenic carbon produced in the soil. About 26% of the samples belong to the deep CO_2_ population with the heavier values of *δ*^13^C_CO2,e_ (*δ*^13^C_CO2,*deep*_ = − 1 ± 0.7‰). The remaining 44% of the samples consist of a mixed population with values of *δ*^13^C_CO2,e_ (− 17 ± 9‰) intermediate between the biogenic and deep CO_2_ populations.

The mean *δ*^13^C_CO2_ value of the deep CO_2_ population (*δ*^13^C_CO2,*deep*_ = − 1 ± 0.7‰), mostly defined by the *δ*^13^C_CO2_ of the fumarolic fluids and by the *δ*^13^C_CO2,e_ of samples C, is significantly heavier than that of the typical magmatic CO_2_ (MORB *δ*^13^C_CO2_ ≈ − 4 to – 8‰ (ref. ^22^ and refs therein)). This divergence may result from a variety of mutually inclusive processes, such as (1) release of crustal CO_2_ from the subducted carbonate, (2) fractionation within the hydrothermal system (e.g., boiling, fluid-rock interaction, precipitation of carbonate minerals), (3) thermal and/or metamorphic decarbonation of the limestone (Aegean limestone *δ*^13^C = − 0.5 to 2.5‰^23,24^) basement of Nisyros (as was suggested also for Santorini volcano^10,25^).

We therefore conclude that the *deep*CO_2_ flux (1) is equals to the *meas*CO_2_ flux (i.e., *bio*CO_2_ flux = 0) for the samples with *δ*^13^C_CO2,e_ > − 1.7 ‰ (i.e., heavier than the mean *δ*^13^C_CO2,*deep*_ − 1σ), and (2) is equals to 0 (i.e., *bio*CO_2_ flux = *meas*CO_2_ flux) for samples with *δ*^13^C_CO2,e_ < − 25.8 ‰ (i.e., lighter than the mean *δ*^13^C_CO2, *bio*_ + 1σ). For the samples with intermediate values of *δ*^13^C_CO2,e_, we computed the fractions of the deep (*Y*) and biogenic (1–*Y*) CO_2_, using the following carbon isotopic mass balance:

1 $$\delta^{13} {\text{C}}_{{\text{CO2, e}}} = \delta^{13} {\text{C}}_{{{\text{CO2}},deep}} \times Y + \delta^{13} {\text{C}}_{{{\text{CO2}},bio}} \times \left( {1{-}Y} \right);$$

Through Eq. (1), we have derived the *deep*CO_2_ flux (*deep*CO_2_ flux = *meas*CO_2_ flux × *Y*) and the *bio*CO_2_ flux (*bio*CO_2_ flux = *meas*CO_2_ flux × (1–*Y*)).

Results show a relatively high *bio*CO_2_ flux estimated for both the samples A and B (Table 1 and Fig. 2), with a mean of 17.6 g m^−2^ d^−1^ and 24.5 g m^−2^ d^−1^, respectively. In particular, the CO_2_ diffusively emitted from the sites of samples B derives mostly from biogenic activity, indicative of a minimal *deep*CO_2_ contribution (Fig. 2). The mean value of *bio*CO_2_ flux of samples B, i.e. 24.5 g m^−2^ d^−1^ (95% confidence interval of 13.3–35.7 g m^−2^ d^−1^), is up to one order of magnitude higher than the mean of the biogenic fluxes estimated for the same area in October 2018 and in 1999–2001 (~ 4 g m^−2^ d^−1^ and 8 g m^−2^ d^−1^, respectively; ref.^18,19,21^). The environmental factors (e.g., air–soil temperature and humidity, rain, wind speed) can affect the biogenic soil CO_2_ emission^26-30^ by favoring soil respiration processes (i.e., roots, microbial and fauna respiration, and decomposition of organic matter^31^). Indeed, the *bio*CO_2_ flux increase is clearly related to the wet meteorological conditions encountered before and during the survey, which promoted growth of vegetation in the Lakki plain. In previous surveys (1999–2001^18^ and 2018^19^), this area was instead characterized by a few shrubs growing in bare soils, due to the very dry summer.

#### Radon and thoron activities and exhalation rates

Given the short half-life (55.6 s) and low recoil range (30 nm) of ^220^Rn, the relatively high activity of ^220^Rn (and low ^222^Rn/^220^Rn ratio) in samples A and B (Fig. 2), measured with RAD7 (see “Methods”), suggests that the nuclides are recoiled in the soil matrix or, at least, at shallow levels (e.g., ^32^). Considering the medium-to-high permeability of the soil (Table 1) and a subsurface fluid flow timescale of ~ 5 min (i.e., five half-lives of ^220^Rn), it is reasonable to conclude that the radon gas measured traveled no further than a few tens of centimeters of depth by diffusion (~ 10^–6^ m^2^ s^−1^^33^). On the other hand, the high activity of ^222^Rn of samples A (see the outliers of ^222^Rn and ^222^Rn/^220^Rn boxplots in Fig. 2) is indicative of a deep contribution by an advective gas-carrying fluid transport^9,13,34-40^. Consistent with this scenario, these samples display also relatively high *deep*CO_2_ flux (see also *deep*CO_2_ flux outliers of samples A in Fig. 2).

The ^222^Rn/^220^Rn ratios of the soil gas samples C are very high and define a separate data population (Fig. 2). The low levels of ^220^Rn of samples C (Fig. 2) are mostly related to a low number of free-state radon atoms residing and accumulating within the altered soil matrix. The persistent and intense circulation of hot, acidic hydrothermal fluids led to argillic alteration and secondary mineralization in this area of the caldera^41^. Accordingly, the soil structure alternates between porous- and sealed portions. The self-sealing of the pore volume between minerals increases the probability of radon recoil into the same or adjacent mineral grains rather than into the pore space^42^, therefore, it locally reduces the emanation of radon^12,43^. Conversely, most of the ^222^Rn activity measured in samples C is of deeper origin and related to the effect of an advective carrier gas (Fig. 2). The absence of correlation (R^2^ = 0.06) between ^222^Rn activity concentrations measured during the survey and the exhalation rate (E_222_) determined in laboratory (see “Methods”), and the extremely low values of E_222_ of both soils and rhyolitic-rhyodacitic rocks (Table 2), strengthen the hypothesis that a large amount of ^222^Rn reaches the surface by an advective transport mechanism controlled by *deep*CO_2_ fluxes. This is particularly evident for the samples C (Fig. 2), which, together with the outliers of samples A, show maximum *deep*CO_2_ fluxes (up to 208.6 g m^−2^ d^−1^) with respect to the entire data set.

**Table 2:** Soil and rock samples used for the determination of the ^222^Rn and ^220^Rn exhalation rates.

	SA7	SA24	SC2	RD	UP
^222^Rn exhalation rate (Bq m^−2^ h^−1^)	0.238 ± 0.056	0.190 ± 0.071	0.126 ± 0.055	0.023 ± 0.016	0.011 ± 0.010
^220^Rn exhalation rate (Bq m^−2^ h^−1^)	379 ± 114	386 ± 116	141 ± 45	29 ± 17	42 ± 37

SA7 refers to a soil sample dominated by shallow source of gas (see A7 in Table 1), SA24 to a soil sample located in an anomalous zone of deep degassing (see A24 in Table 1) and SC2 to a soil collected from the active hydrotherml area of the caldera (see C2 in Table 1). RD refers to a rhyodacitic lava of the post-caldera domes and UP refers to a rhyolitic pumice from the Upper Pumice.

### Deep versus shallow degassing component and DDS definition

**Figure 6:**
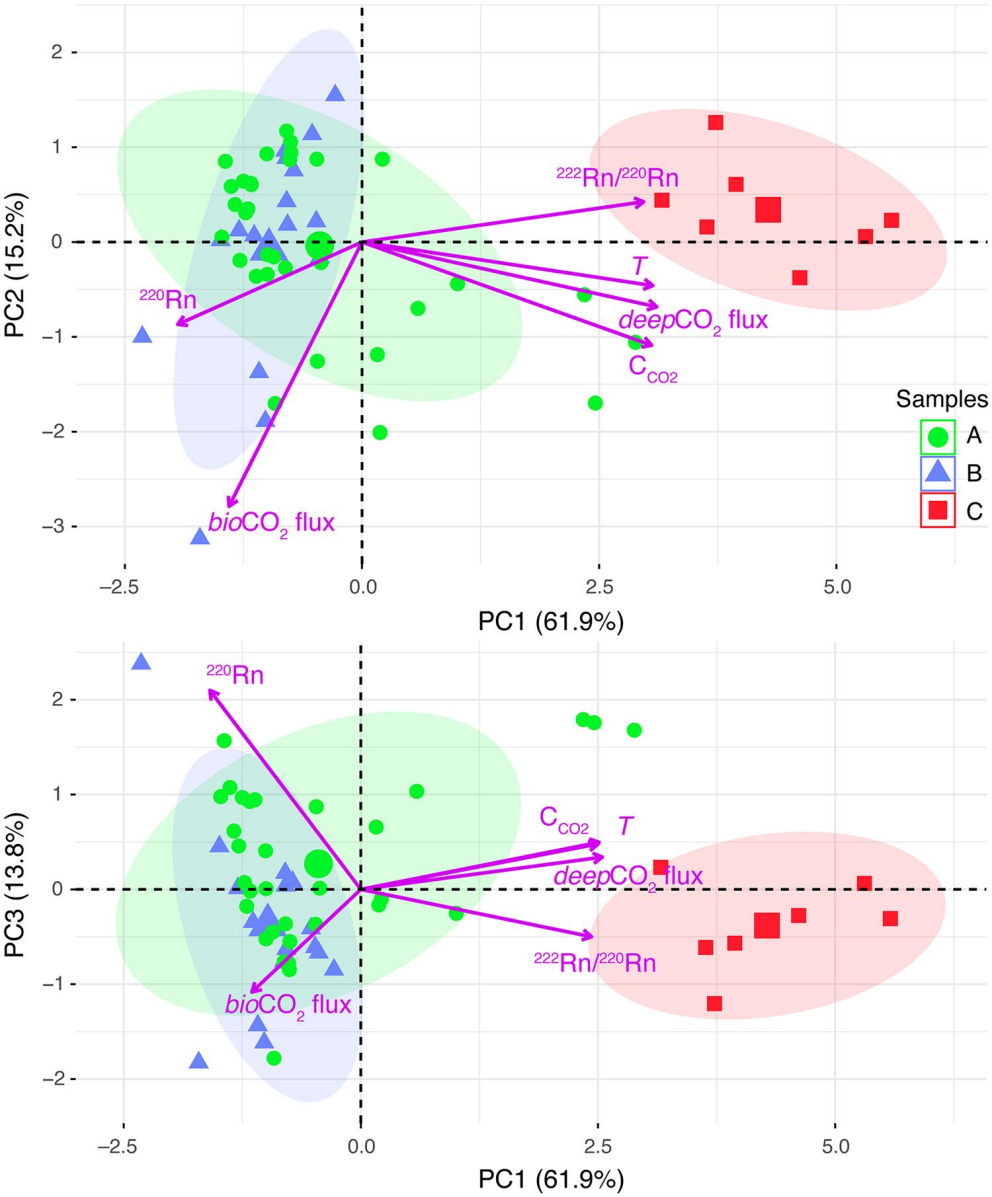
Biplot of PC1 versus PC2 and PC1 versus PC3 for the data acquired on April 2019. The markers represent the scores of samples A, B, and C on the PC1, PC2, and PC3 extracted from Principal Component Analysis. The purple arrows indicate the PC loading vectors. The length of the vector from the origin reflects the variance of the variable. The correlation between two variables is given by the angle between two vectors; the smaller is the angle, the greater is the correlation^65^. For each group of samples, 80% bivariate ellipses of the scores are drawn.

Despite careful inspection of the scatterplot matrix (Fig. S2), there is no straightforward way to interpret correlations between variables, in particular to distinguish whether they are controlled by deep or shallow degassing processes. To simplify the interpretation, we investigate which variable, or combination of variables, control the observed large (spatial) variability of the data. Thus, we performed a Principal Component Analysis (PCA, see “Methods”) on the different measured parameters (*deep*CO_2_ flux, *bio*CO_2_ flux, *T*, C_CO2_, ^220^Rn, and ^222^Rn/^220^Rn ratio). PCA aims at defining a set of linearly uncorrelated variables called principal components (PC), ranking them in terms of their overall control on the variance. Therefore, PCA, which is often used to reduce dimensionality in the data set by selecting those variables that mainly control the variance in the data, is here applied to summarize and simplify the relationships among the presented multivariate set of data. In our case, the firsts three PC cumulatively retain 90.9% of the total variance in the data. The eigenvectors matrix and the importance of components in Table 3a show that PC1 explains the 61.9% of the variance in the data set, and describes a dimension to which *deep*CO_2_ flux, C_CO2_, *T*, and ^222^Rn/^220^Rn ratio contribute almost equally. PC2 retains the 15.2% of the variance in the data set and delineates a dimension mainly correlated with *bio*CO_2_ flux. PC3 accounts for 13.8% of the variance in the data set, suggesting that most of the contribution is loaded by ^220^Rn and *bio*CO_2_ flux. The remaining PC (i.e., PC4, PC5, and PC6) explain only a minor part of the total variance in the data and, hence, are not further considered. Such results from PCA clearly indicate that the variance in the data is governed by two different degassing components: (1) PC1 is related to deeper sources and (2) PC2 and PC3 (i.e., the residual variance) are related to shallower processes. This is particularly evident upon inspection of the biplots (Fig. 6), displaying both the principal component scores (i.e., circles, triangles, and squares for samples A, B and C, respectively) and the loading vectors (i.e., purple arrows). PC1 places much weight on variables strongly controlled by magmatic-hydrothermal processes (i.e., *deep*CO_2_ flux, *T*, C_CO2_, and ^222^Rn/^220^Rn ratio). Their almost equal contribution on PC1 is documented by the length of each vector (Fig. 6), while their strong positive correlation is shown by the low angles between the vectors (Fig. 6). The shallow degassing component (represented by PC2 and PC3) depends on *bio*CO_2_ flux and ^220^Rn activity, which are quite positively correlated in PC2, and uncorrelated in PC3 (Fig. 6). The absence of a clear correlation attests to the independent origins of the two shallow signals: *bio*CO_2_ flux is controlled by soil biogenic processes, while ^220^Rn activity depends on the physical–chemical properties of the soil (e.g., Ra content in the mineral phase and porosity).

**Table 3:** Principal Component Analysis (PCA) of *deep*CO_2_ flux, *bio*CO_2_ flux, C_CO2_, *T*, ^222^Rn/^220^Rn ratio, and ^220^Rn activity. (a) refers to the PCA performed using the whole data set; (b) refers to the PCA performed using the data of samples A (Table 1). “Propor. Variance” indicates the amount of the variation represented by the different eigenvalues. “Cum. Prop.” indicates the proportion of variance cumulatively retained by the eigenvalues.

(a)	Eigenvectors					
Variables	PC1	PC2	PC3	PC4	PC5	PC6
*deep*CO_2_ flux	0.4743	− 0.2103	0.1343	− 0.1701	0.6914	0.4537
*bio*CO_2_ flux	− 0.2140	− 0.8574	− 0.4274	− 0.1003	− 0.1243	0.1046
C_CO2_	0.4660	− 0.3349	0.1890	0.1420	0.0879	− 0.7791
*T*	0.4679	− 0.1414	0.1961	0.4937	− 0.5533	0.4157
^222^Rn/^220^Rn	0.4531	0.1307	− 0.1973	− 0.7564	− 0.4078	− 0.0169
^220^Rn	− 0.2965	− 0.2672	0.8284	− 0.3535	− 0.1624	0.0557
**Importance of components**						
Eigenvalue	3.7140	0.9144	0.8281	0.2810	0.1729	0.0896
Propor. Variance	0.6190	0.1524	0.1380	0.0468	0.0288	0.0149
Cum. Prop	0.6190	0.7714	0.9094	0.9563	0.9851	1.0000

(b)	Eigenvectors					
Variables	PC1	PC2	PC3	PC4	PC5	PC6
*deep*CO_2_ flux	0.4952	0.0297	− 0.0297	− 0.5872	− 0.6388	0.0115
*bio*CO_2_ flux	0.0040	0.7243	0.6672	0.1081	− 0.0917	0.1002
C_CO2_	0.5126	0.0367	0.1154	0.0359	0.3468	− 0.7753
*T*	0.5119	0.0071	0.0125	− 0.1966	0.5880	0.5945
^222^Rn/^220^Rn	0.4749	0.0125	− 0.2120	0.7701	− 0.3263	0.1727
^220^Rn	0.0670	− 0.6877	0.7039	0.1023	− 0.1055	0.0740
**Importance of components**						
Eigenvalue	3.6426	1.1589	0.8724	0.2202	0.0711	0.0347
Propor. Variance	0.6071	0.1932	0.1454	0.0367	0.0119	0.0058
Cum. Prop	0.6071	0.8003	0.9457	0.9824	0.9942	1.0000

PC1, PC2, and PC3 displayed in Fig. 6 satisfactorily reproduce the geochemical differences observed among samples A, B and C. Samples with large positive scores on PC1 are highly influenced by a deep degassing process, whereas those with negative scores are weakly dependent on hydrothermal system dynamics. The analysis indicates that samples C are most affected by the deep component (Fig. 6), in agreement with the very active hydrothermal circulation of fluids in this portion of the caldera, whereas samples B are mostly affected by the shallow soil activity. Consequently, their scores are located in a narrow portion of the negative PC1 dimension, while they have significantly higher scores on PC2 and PC3 (Fig. 6). On the other hand, samples A appear to reflect contributions from both deep and shallow degassing components. A consistent part of the scores overlaps in the region of shallow degassing defined by the scores of samples B, while the remaining scores of samples A spread over the PC1 dimension and approach the values of samples C (Fig. 6). The overall distribution of the samples A, intermediate between those of samples B and C, is distinguished by the shape and orientation of the green ellipses.

**Figure 7:**
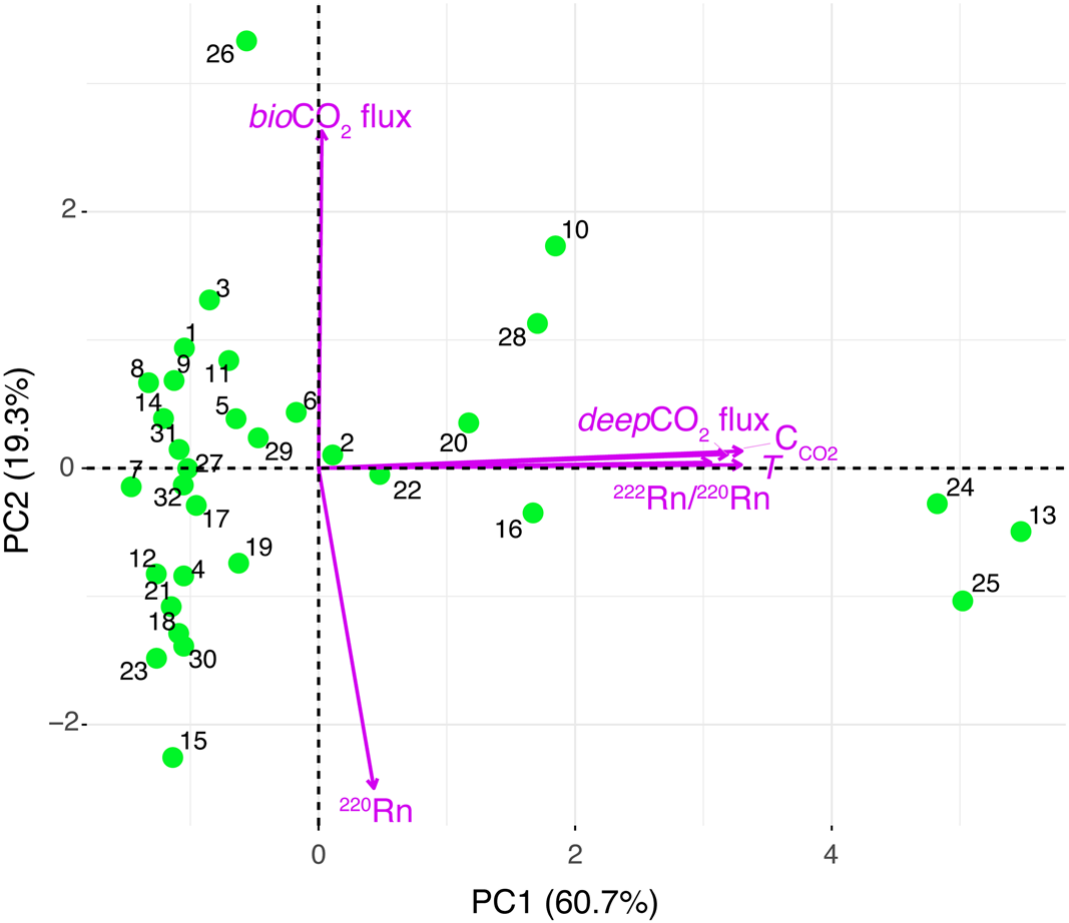
Biplot of PC1 versus PC2 for the samples A data acquired on April 2019. The numbers associated to the green circles (namely, the scores of PC1 and PC2) correspond to the number of samples A in Table 1. The very low angles between the *deep*CO_2_ flux, *T*, C_CO2_, and ^222^Rn/^220^Rn ratio loading vectors indicate a strong positive correlation between these variables, which together define the deep degassing component PC1.

The distinction between a deep and a shallow degassing component becomes particularly clear from analysis of PCA results performed using only samples A (Table 3b; Fig. 7). Notably, 94.6% of the variance in the data can be explained by PC1 (60.7%), PC2 (19.3%) and PC3 (14.5%). PC1 is almost entirely controlled by *deep*CO_2_ flux, C_CO2_, *T*, and ^222^Rn/^220^Rn ratio (Table 3b), to which they contribute almost equally, and they are near-perfectly positively correlated (Fig. 7). PC2 and PC3 are strongly controlled by *bio*CO_2_ flux and ^220^Rn (with an almost equal contribution; Table 3b), and they are negatively correlated (Fig. 7).

The data sets of the four variables defining the deep degassing component were processed to construct *deep*CO_2_ flux, C_CO2_, *T*, and ^222^Rn/^220^Rn ratio 2D maps of the DDS extending over the site from which samples A were taken, by a sequential Gaussian simulation (sGs, see “Methods”). These maps are compared to the CO_2_ flux map in order to understand differences in the estimation of the degassing from DDS (spatial distribution and quantitative estimation of the gas output) deriving from the multi-parametric approach.

**Figure 8:**
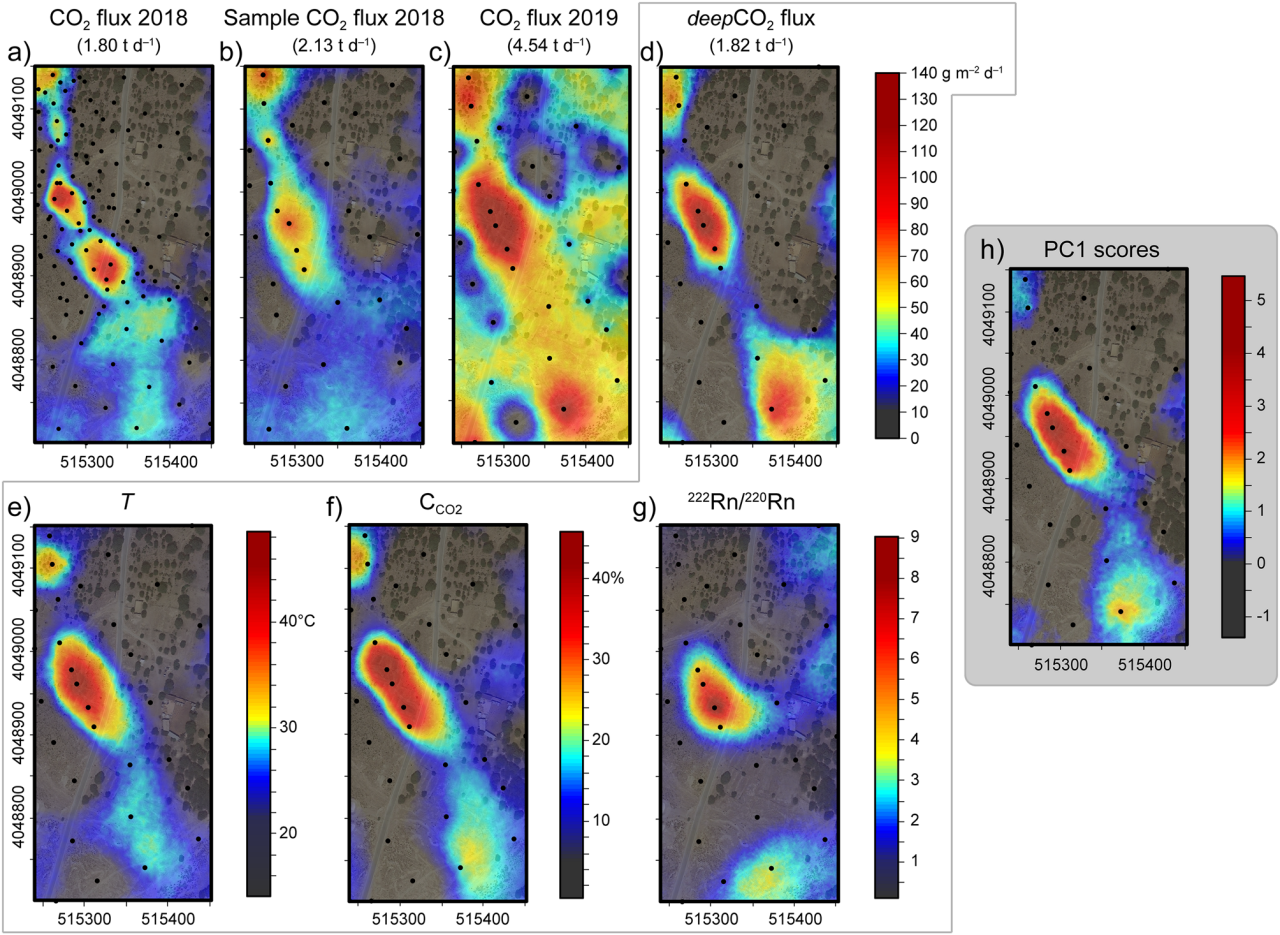
E-type maps constructed by a pointwise linear average of 200 sequential Gaussian simulations of different attributes over the area covered by samples A. The black circles represent the sampling sites. The maps show: (**a**) the CO_2_ flux measured during October 2018^19^. (**b**) the CO_2_ flux measured during October 2018, produced using 32 points sampled from the original data set. (**c**) the CO_2_ flux measured on April 2019. (**d**) the *deep*CO_2_ flux. (**e**) *T*. (**f**) C_CO2_. (**g**) ^222^Rn/^220^Rn ratio. (**h**) PC1 scores. The maps were created with the software Surfer, version 11.0.642 (https://www.goldensoftware.com/products/surfer). Easting and northing coordinates refer to the WGS 84/UTM zone 35 S.

The map of the NW–SE alignment of the DDS highlighted during the previous campaign (Fig. 8a), shows the spatial distribution of the CO_2_ fluxes measured in October 2018^19^. The maps of *T* (Fig. 8e) and C_CO2_ (Fig. 8f) in this region are very well correlated, and the spatial distribution of the anomaly is in concordance with the 2018 CO_2_ flux alignment. The new map of CO_2_ fluxes recorded in April 2019 (Fig. 8c) follows the general trend of outgassing from NW towards SE but, at the same time, illustrates a large extension of the DDS. Furthermore, the total CO_2_ output of the DDS in April 2019 is estimated at 4.54 t d^−1^, while the same area in October 2018, when the *bio*CO_2_ flux was very low, emitted 1.80 t d^−1^. Our new *deep*CO_2_ flux data from April 2019 (Fig. 8d) provide an estimation of the total output of CO_2_ (1.82 t d^−1^) very close to that found on October 2018, thereby excluding the possibility of any change in hydrothermal-volcanic CO_2_ emission. This conclusion is also supported by the absence of any manifestations (e.g., earthquakes, ground deformations, gravimetry changes, and increase of fumarolic activity) of volcanic unrest recorded after October 2018, that instead occurred at Nisyros after the seismic crisis of the 1996–1997^18,20,44-48^. Nevertheless, the October 2018 map of CO_2_ flux was constructed using more points (i.e., 124) than those collected in the April 2019 survey. To facilitate comparison between the two data sets, we used the October 2018 data set to randomly draw a set of measurements (i.e., 32) identical of those from the April 2019 data set, following their spatial distribution as closely as possible (Fig. 8b). Obviously, the DDS loses detail on a graphical representation, but the new estimation for the CO_2_ output of October 2018 is still less than the half of April 2019 (i.e., 2.13 t d^−1^ versus 4.54 t d^−1^), and hence the discrepancy in CO_2_ fluxes between the two dates cannot be explained by a difference in sampling density.

Therefore, the CO_2_ flux measurements alone would lead to the erroneous conclusion that the increased extent and total CO_2_ output from the DDS resulted from magmatic-hydrothermal activity. Instead, because the biogenic CO_2_ production strongly increased in this area in April 2019, accounting for about 60% (~ 2.72 t d^−1^) of the total CO_2_ daily output, the total CO_2_ flux also increased, despite the fact that the intensity of the volcanic activity was similar at the time of the two surveys. This behavior can be quantified with estimates of the *deep*CO_2_ flux, which more faithfully tracks the extent of the DDS (Fig. 8d) and its total CO_2_ output. Importantly, the *deep*CO_2_ flux map pairs with the anomaly defined by the ^222^Rn/^220^Rn ratio map (Fig. 8g), suggesting again the advective transport of ^222^Rn by *deep*CO_2_. More generally, the extent of the DDS defined by the variables linked to the deep degassing (*deep*CO_2_ flux, *T*, C_CO2_, and ^222^Rn/^220^Rn ratio) are very well correlated with each other (Fig. 8d–g). The map of the PC1 scores (Fig. 8h), i.e. the deep degassing component returned from PCA, integrates all information from *deep*CO_2_ flux, *T*, C_CO2_, and ^222^Rn/^220^Rn ratio maps, thereby most accurately reflecting the distribution of magmatic-hydrothermal outgassing relative to shallow background gases. The importance of the multi-parametric approach is underlined by the capacity to better define the spatial distribution of DDS.

## Concluding remarks

The multi-parametric approach presented in this study has important ramifications for a better understanding of the degassing behavior of magmatic-hydrothermal systems. Correct interpretation of diffuse CO_2_ fluxes from the soil is not a trivial task in active volcanic areas, especially when measurements are conducted in different periods of the year affected by seasonality and/or in humid and vegetated areas. The Nisyros caldera represents an ideal test site in which seasonal variations may result in changes in biogenic gas production with respect to the magmatic-hydrothermal CO_2_ emissions. We demonstrate that the biological processes contribute up to 60% of the total CO_2_ output, thus causing potential misinterpretation of surveillance measurements of CO_2_ fluxes if they were to be attributed to volcanic-hydrothermal activity alone. The analysis of different soil gas parameters (*δ*^13^C_CO2,_ C_CO2_, *T*, and ^222^Rn/^220^Rn ratio) permits the classification and interpretation of the different contributors to the observed hydrothermal gas emission. Through a Principal Component Analysis, we identify three main components. The component that predominantly controls the variance (PC1) is correlated to a deep degassing process, which is closely associated with hydrothermal system dynamics (i.e., *deep*CO_2_ flux, *T*, C_CO2_, and ^222^Rn/^220^Rn ratio); the other significant components (PC2 and PC3) are instead related to shallow degassing processes (i.e., *bio*CO_2_ flux and ^220^Rn). Hence, the main result from the PCA is to integrate all indicators of deep gas sources into one component not affected by shallow, non-hydrothermal processes (e.g., seasonal/biological effect on gas production).

## Methods

### Field survey and laboratory analysis

#### Diffuse CO_2_ flux and isotopic compositions of the CO_2_ efflux

Following ref.^11^, the diffuse emission of CO_2_ from the soil was measured with the accumulation chamber (AC) methodology, employing an instrument developed at the Università di Perugia and described in detail in ref.^19^. The gas line from the infrared sensor (IR) to the AC, was modified by inserting a T-connector with a pierceable septum which permits direct sampling of the gas phase^2,10,49^ (see Fig. 2 in ref.^2^). During each flux measurement, two samples of gas were collected using a syringe equipped with a shut-off valve and then stored in a 12 mL-evacuated-vial (Labco Exetainer) for further analysis of *δ*^13^C_CO2_ and CO_2_ concentration. The first sample was taken at the beginning of the AC measurement when the CO_2_ concentration was relatively low, while the second sample was taken later when the CO_2_ concentration increased (see samples C_CO2,I_ and C_CO2,II_, respectively, in Table S1). The carbon isotopes of CO_2_ (*δ*^13^C_CO2,I_ and *δ*^13^C_CO2,II_, Table S1) were determined within a week of the sampling at the laboratories of INGV Osservatorio Vesuviano. The samples were analyzed using a continuous flow isotope ratio mass spectrometer (Thermo-Finnigan Delta XP) interfaced with a Gasbench II device that was equipped with an autosampler (*δ*^13^C_CO2_ standard error ± 0.1‰). For each sample, CO_2_ concentrations were determined both in the field (using the IR of the AC) and in laboratory together with the isotopic analysis. The determined concentrations were found to be in good accord, demonstrating the accuracy of the two techniques (Fig. S1).

#### Isotopic composition of the fumarolic CO_2_

During the survey, the main fumaroles of Lakki plain were sampled and analyzed (Table S2) to have an independent measurement of *δ*^13^C_CO2_ of deep origin involved in the diffuse degassing process. The used sampling and analytical methods employed are extensively described in ref.^50^.

#### ^222^Rn-^220^Rn activity, soil pressure–temperature and CO_2_-H_2_S concentration

Soil gas measurements of ^222^Rn (radon, with half-life of 3.82 days) and ^220^Rn (thoron, with half-life of 55.6 s) nuclides at Nisyros caldera were performed using the RAD7 monitoring system (Durridge Company Inc., USA). The setup is equipped with a solid-state ion-implanted planar silicon detector, a pump with a flow rate of 1 L min^−1^, a gas-drying unit filled with a desiccant (CaSO_4_ with 3% CoCl_2_, as indicator) and an inlet filter (pore size 1 µm) for the fine dust particles. The factory-calibrated detector operates in a sensitivity range of 4–80,000 Bq m^-3^, with an accuracy of 5%. The soil gas measurement was carried out in “sniff mode” by determining ^222^Rn and ^220^Rn concentrations from the energy windows of 5.40–6.40 and 6.40–7.40 MeV, thus detecting the total counts (at 6.00 MeV) of alpha particles from the 3.04-min ^218^Po decay (^222^Rn daughter) and the total counts (at 6.78 MeV) of alpha particles from the 0.145-s ^216^Po (^220^Rn daughter). A stainless-steel gas probe (manufactured by RADON v.o.s. Inc.) was inserted into the soil at depth of 80 cm with the aid of a hammer and then connected to the inlet of the RAD7 via vinyl tubing. A small cylindrical cavity was created just below the probe head by the extrusion of a lost tip. The volume of the cavity (about 5.6 cm^3^) was large enough to enable soil gas collection using the RAD7 built-in pump. After a purging time of 10 min, the alpha particles were collected by a measurement cycle of 15 min. When the terrain is very humid, the water content in RAD7 gradually increases, even if a desiccant is employed. The radon activity concentration was progressively underestimated because of neutralization processes affecting radon daughters during electrostatic collection. The radon signal was, therefore, corrected according to the methodological approach reported in ref.^51^.

Soil gas permeability was also obtained by using PRM3 permeameter^52^. The instrument draws air from the same hollow probe used for radon measurement. The permeameter is equipped with a pump and a vacuum gauge that reads the negative pressure (*ΔP*), induced by soil gas extraction through the terrain. Intrinsic permeability is calculated according to a modified version of Darcy’s equation where the air flow (*Q*) is replaced by a linear equation of the form: *Q* = (*m* × *ΔP* + *c*), where m and c are the slope and the intercept of the instrument calibration curve, respectively.

Soil temperature and CO_2_-H_2_S concentration were measured with a K-type thermocouple and a Dräger X-am 7000 analyzer (Drägerwerk AG & Co. KGaA Inc., Germany), respectively. This latter instrument is equipped with an infrared sensor (full-scale 100% in volume, sensibility of 0.2%) for CO_2_ measurement, and with an electrochemical cell (full-scale 500 ppm, sensibility of 0.5 ppm) for H_2_S measurement. Since H_2_S was used to select sampling points with very low sulfur concentrations and generally close to 0%, its value is not reported in this study.

#### ^222^Rn-^220^Rn exhalation rate in laboratory

In order to determine the exhalation rate of ^222^Rn-^220^Rn in laboratory, several rock and soil samples from Nisyros caldera were also collected: (1) a rhyolitic pumice belonging to the Upper Pumice succession (sample UP), one of the caldera-forming Plinian eruptive cycles^53,54^, (2) a rhyodacitic lava (sample RD) from the post-caldera domes (in particular, the small dome of Lofos), following the Upper Pumice eruption^55,56^, (3) the soil from site A7 (sample SA7), (4) the soil from site A24 (sample SA24), and (5) the soil from site C2 (sample SC2). The closed-loop experimental setup is described in ref.^57,58^ and briefly consists of a stainless-steel accumulation chamber (5.1 L) connected via vinyl tubing to a gas-drying unit and to the RAD7. The accumulation chamber is immersed into a refrigerating thermostatic bath and kept at the constant temperature of 30 °C to avoid the oscillation of the radon activity concentration under the effect of thermal gradients. The detection limit of the experimental apparatus is equal to 0.01 and 6 Bq h^−1^ for ^222^Rn and ^220^Rn, respectively, provided that the activity concentration was corrected for the humidity and temperature measured by the radon detector (see ref.^57,58^). The duration of one single measurement was 24 h and ^222^Rn-^220^Rn mass exhalation rates were calculated for pre-dried samples (in an oven for 24 h) through the following equations:

2 $$E_{222} = \left( {m + \lambda_{222} \times C_{222} } \right) \times V$$

and

3 $$E_{220} = \lambda_{220} \times V_{0} \times \frac{{C_{222} }}{{e^{{({-}\lambda_{220} \times \frac{{V_{1} }}{Q})}} }}$$

where *E*_222_-*E*_220_ (Bq m^−2^ h^−1^), *C*_222_-*C*_220_ (Bq m^-3^), and *λ*_222_-*λ*_220_ (h^−1^) are mass exhalation rates, activity concentrations and decay constants of ^222^Rn and ^220^Rn, respectively. *V*, *V*_0_*,* and *V*_1_ (m^3^) are the free total volume of the system, the volume of the accumulation chamber and the volume of the vinyl tubing, respectively. *Q* (m^3^ h^−1^) is the pump capacity and *m* (Bq m^-3^ h^−1^) is the initial slope of the ^222^Rn growth curve.

### Statistical analysis

#### Graphical statistical approach (GSA)

The polymodal distribution of the data results from the presence of more populations of data within the same distribution. In soil gas measurements, the occurrence of two or more populations can be related to the presence of multiple geochemical processes/sources controlling the observed variable. The GSA^11^ aims to identify and define each population of data in such a polymodal distribution. The approach consists of plotting the data on a probability plot, where a normal population plots on a straight line, whereas a polymodal distribution of *n* normal populations defines a curve with *n*−1 inflection points. Using a graphical procedure^59,60^, it is possible to subdivide such complex statistical distributions into individual normal populations and compute the fraction, the mean and the standard deviation of each of them. Since the computed means for the soil *T* and C_CO2_ refer to the logarithm of the *T* and C_CO2_ values, the mean values of such variables were then estimated using a Monte Carlo simulation procedure.

#### Principal component analysis (PCA)

PCA is a dimension reduction technique that allows the derivation of a low-dimensional set of components from a large *n* × *p* data matrix^61^. Strictly speaking, with a small number of principal components one can summarize most of the information of the original data set, composed by a large number of variables *p* and observations *n*. The principal components (PC) are a set of linearly uncorrelated variables, ranked in terms of their overall control on the variance. Along the first principal component (PC1) direction the data vary the most, and so on with a progressive decreasing in the variability of the data for the remaining principal components. Hence, the first three PC (in our case) collectively explain most of the variance in the set of data. PCA was performed initially on the whole data set (composed by *deep*CO_2_ flux, *bio*CO_2_ flux, *T*, C_CO2_, ^222^Rn/^220^Rn ratio, and ^220^Rn activity) and secondly only on samples A data. PCA was performed using the function *prcomp* of the package *stats*^63^ of the R statistical software^63^ on scaled variables. Results are then visualized using the package *factoextra*^64^.

#### Sequential Gaussian simulation (sGs)

In order to visualize the spatial distribution of different attributes (i.e., the variables considered in the PCA) over the area of samples A, we produced 2D E-type maps using the conditional sequential Gaussian simulation (sGs). The stochastic simulations were performed using the algorithm of *sgsim* code^62^, and considering as attributes *deep*CO_2_ flux, *T*, C_CO2_, ^222^Rn/^220^Rn ratio, and PC1 scores. The variables were simulated at each unsampled location defined by a regular grid of 107 × 226 cells of 2 m × 2 m (covering the samples A area), to reproduce the statistical and spatial distribution of each attribute (i.e., the semivariogram of the normal scores of the variable). The values are randomly drawn from a Gaussian conditional cumulative distribution function, which depends on the original data and on the data previously simulated^21^. The simulations were run in order to produce 200 equiprobable realizations for each data set. The E-type maps of the variables considered were derived through a pointwise linear average of all the realizations.

## Supplementary information

Supplementary Information.
