# Supplementary material for: Deep versus shallow sources of CO2 and Rn from a multi-parametric approach: the case of the Nisyros caldera (Aegean Arc, Greece)
Source: Sci Rep. 2020 Aug 13;10:13782. doi: 10.1038/s41598-020-70114-x (PMC7426811; doi:10.1038/s41598-020-70114-x)
Supplement: Supplementary file 1 — Supplementary Information. [file 41598_2020_70114_MOESM1_ESM.pdf]

Supplementary Information

Deep vs. shallow sources of CO_2 and Rn from a multi-parametric approach: the case of the Nisyros caldera (Aegean Arc, Greece)

Giulio Bini[1], Giovanni Chiodini[2], Carlo Lucchetti[3], Piergiorgio Moschini[3], Stefano Caliro[4], Silvio Mollo[3,5], Jacopo Selva[2], Paola Tuccimei[6], Gianfranco Galli[5], and Olivier Bachmann[1]

[1] Institute of Geochemistry and Petrology, Department of Earth Sciences, ETH Zurich, 8092, Switzerland
[2] Istituto Nazionale di Geofisica e Vulcanologia, Sezione di Bologna, 40128, Italy
[3] Dipartimento di Scienze della Terra, Sapienza-Università di Roma, Rome, 00185, Italy
[4] Istituto Nazionale di Geofisica e Vulcanologia, Osservatorio Vesuviano, Sezione di Napoli, 80124, Italy
[5] Istituto Nazionale di Geofisica e Vulcanologia, Sezione di Roma 1, 00143, Italy
[6] Dipartimento di Scienze, Università Roma Tre, Rome, 00146, Italy

Supplementary Figure S1

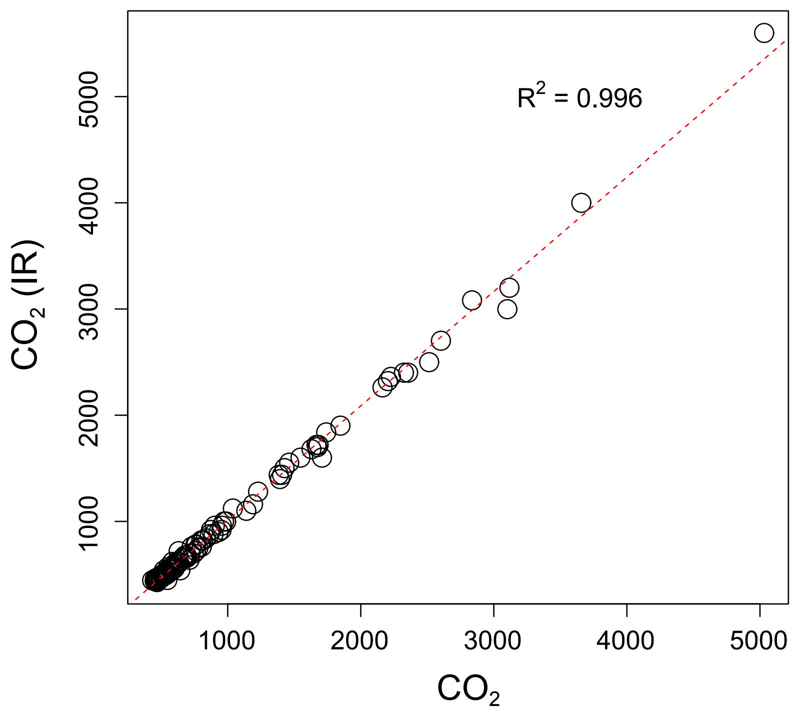

Figure S1. Scatter plot of CO_2 vs. CO_2 (IR). CO_2 refers to the concentration of CO_2 of samples I and II measured in laboratory, while CO_2 (IR) refers to the concentration of CO_2 measured in the field by the infrared sensor of the accumulation chamber, during the collection of samples I and II. Both the concentrations, i.e. CO_2 and CO_2 (IR), are expressed as ppm by volume.

Supplementary Figure S2

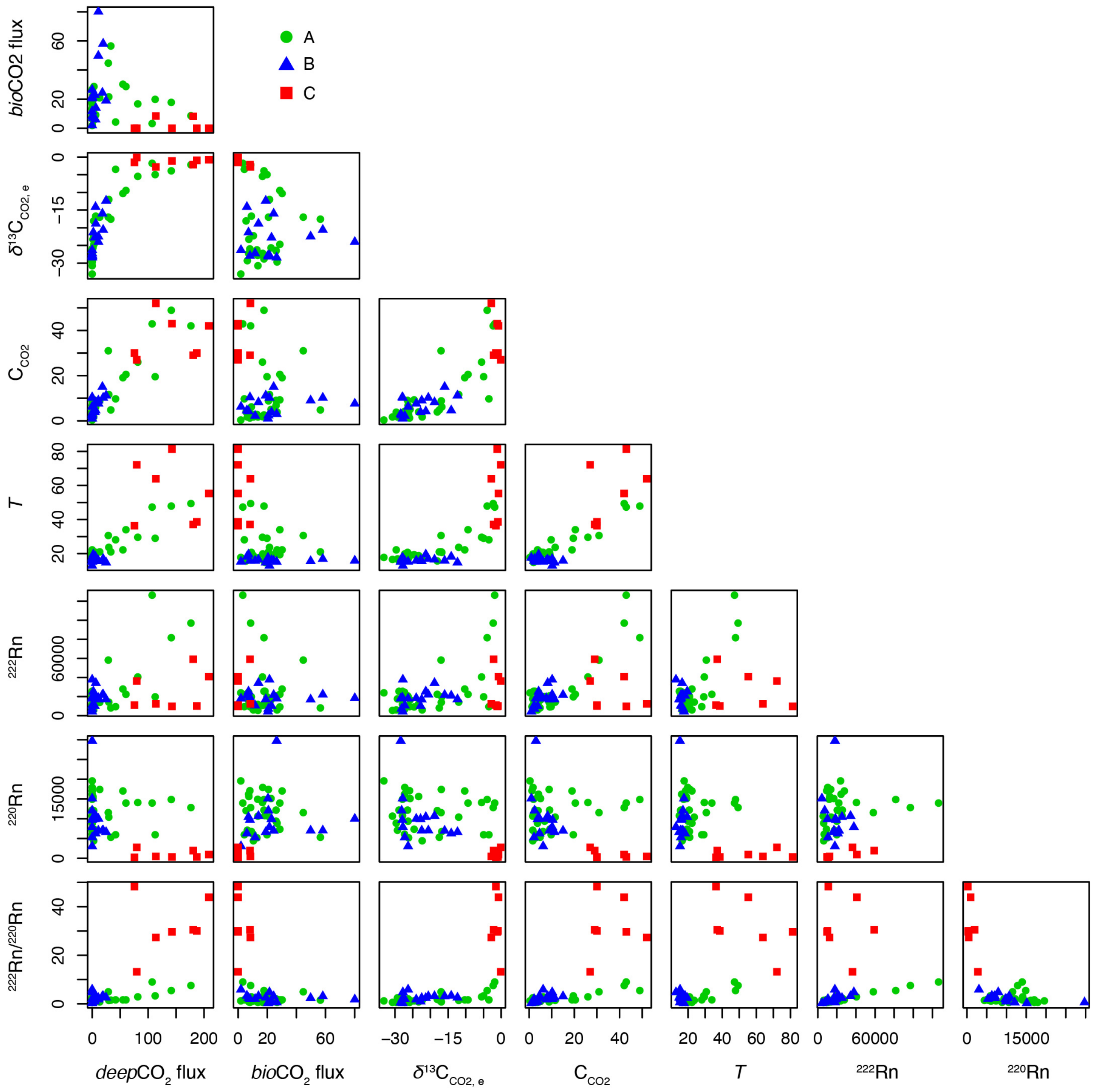

Figure S2. Scatterplot matrix of *deep*CO_2 flux, *bio*CO_2 flux, $\delta^{13}C_{CO2,\,e}$, C_{CO2}, T, ^{222}Rn, ^{220}Rn and ^{222}Rn/^{220}Rn ratio.

Supplementary Table S1. $\delta^{13}C_{CO2}$ and C_{CO2} of the samples of gas inside the AC collected during each CO_2 flux measurement (sample I and sample II).

**Samples I**			**Samples II**		
**n.**	$\delta^{13}C_{CO2, I}$	$C_{CO2, I}$	**n.**	$\delta^{13}C_{CO2, II}$	$C_{CO2, II}$
	‰ vs. PDB	ppm		‰ vs. PDB	ppm
A1_I	-13.55	544	A1_II	-16.69	675
A2_I	-10.49	552	A2_II	-7.89	876
A3_I	-14.31	522	A3_II	-17.24	728
A4_I	-13.08	454	A4_II	-13.61	508
A5_I	-12.48	493	A5_II	-16.34	680
A6_I	-11.57	548	A6_II	-11.72	865
A7_I	-12.88	509	A7_II	-16.17	625
A8_I	-12.30	479	A8_II	-14.27	597
A9_I	-12.30	497	A9_II	-15.85	666
A10_I	-11.59	600	A10_II	-15.05	1683
A11_I	-13.15	526	A11_II	-16.71	721
A12_I	-13.13	511	A12_II	-18.23	781
A13_I	-6.91	904	A13_II	-3.71	2837
A14_I	-12.87	550	A14_II	-14.82	636
A15_I	-10.89	469	A15_II	-14.05	547
A16_I	-8.79	765	A16_II	-7.25	1426
A17_I	-16.51	713	A17_II	-18.86	957
A18_I	-11.45	483	A18_II	-13.82	604
A19_I	-13.93	691	A19_II	-14.62	895
A20_I	-9.96	806	A20_II	-9.68	1674
A21_I	-12.69	509	A21_II	-17.48	758
A22_I	-10.52	761	A22_II	-10.39	1668
A23_I	-12.10	433	A23_II	-17.09	678
A24_I	-7.75	802	A24_II	-5.12	2601
A25_I	-7.29	662	A25_II	-3.44	2204
A26_I	-12.49	585	A26_II	-15.61	1547
A27_I	-10.91	458	A27_II	-15.58	615
A28_I	-7.18	955	A28_II	-5.64	3101
A29_I	-11.26	523	A29_II	-14.33	1191
A30_I	-10.94	474	A30_II	-16.89	745
A31_I	-14.22	585	A31_II	-20.87	1141
A32_I	-13.93	554	A32_II	-19.98	932
B1_I	-11.32	450	B1_II	-13.76	538
B2_I	-12.75	570	B2_II	-18.66	2324
B3_I	-11.73	519	B3_II	-15.10	988
B4_I	-13.02	522	B4_II	-22.91	1463
B5_I	-11.05	460	B5_II	-18.41	815
B6_I	-11.83	484	B6_II	-19.01	1407
B7_I	-13.61	645	B7_II	-15.26	2163
B8_I	-10.64	510	B8_II	-11.81	1631
B9_I	-12.73	561	B9_II	-20.13	2355
B10_I	-13.76	538	B10_II	-21.65	1229
B11_I	-11.47	481	B11_II	-18.17	837
B12_I	-11.95	501	B12_II	-19.80	973
B13_I	-11.26	460	B13_II	-14.59	689
B14_I	-10.78	477	B14_II	-11.79	687
B15_I	-12.28	532	B15_II	-20.62	1846
B16_I	-12.78	521	B16_II	-22.20	1392
C1_I	-7.92	696	C1_II	-4.53	1382
C2_I	-8.35	615	C2_II	-3.03	1739
C3_I	-8.99	604	C3_II	-4.48	2227
C4_I	-4.13	1709	C4_II	-2.42	3659
C5_I	-2.70	3118	C5_II	-1.96	5030
C6_I	-7.52	630	C6_II	-5.15	1039
C7_I	-8.04	628	C7_II	-3.61	2514

Supplementary Table S2. $\delta^{13}C_{CO2}$ in the fumarolic fluids emitted from the hydrothermal area of Nisyros.

Sample	Date	$\delta^{13}C_{CO2}$
		‰ vs. PDB
Kaminakia	04/04/19	-0.81
Ramos	04/04/19	-1.49
Polyvotis Mikros	04/04/19	-0.35
Phlegeton	04/04/19	-0.33
Lofos dome	04/04/19	-0.85
Stefanos 1	05/04/19	-0.98
Stefanos 2	05/04/19	-0.80
Polivotys Mikros Sud	11/04/19	-0.07
Phlegeton 2 Nord	11/04/19	-0.57
